# Caregiver Engagement in Stroke Care: Opportunities and Challenges in Australia and Denmark

**DOI:** 10.3389/fpubh.2021.758808

**Published:** 2021-11-26

**Authors:** Elton H. Lobo, Mohamed Abdelrazek, John Grundy, Finn Kensing, Patricia M. Livingston, Lene J. Rasmussen, Sheikh Mohammed Shariful Islam, Anne Frølich

**Affiliations:** ^1^School of Information Technology, Deakin University, Geelong, VIC, Australia; ^2^Department of Public Health, University of Copenhagen, Copenhagen, Denmark; ^3^Faculty of Information Technology, Monash University, Clayton, VIC, Australia; ^4^Department of Computer Science, University of Copenhagen, Copenhagen, Denmark; ^5^Faculty of Health, Deakin University, Geelong, VIC, Australia; ^6^Department of Cellular and Molecular Medicine, University of Copenhagen, Copenhagen, Denmark; ^7^Center for Healthy Aging, University of Copenhagen, Copenhagen, Denmark; ^8^Institute for Physical Activity and Nutrition (IPAN), Deakin University, Geelong, VIC, Australia; ^9^Innovation and Research Centre for Multimorbidity, Slagelse Hospital, Region Zealand, Denmark

**Keywords:** engagement (involvement), stroke, caregiving—informal, health planning, recovery, policy and guidelines

## Abstract

Globally, there is a rise in incident cases of stroke, particularly in low- and middle-income countries, due to obesity-related and lifestyle risk factors, including health issues such as high cholesterol, diabetes and hypertension. Since the early 20th century, stroke mortality has declined due to proper management of the risk factors and improved treatment practices. However, despite the decline in mortality, there is an increase in the levels of disability that requires long-term support. In countries such as Australia and Denmark, where most care is provided within the community; family members, generally spouses, assume the role of caregiver, with little to no preparation that affects the quality of care provided to the person living with stroke. While past research has highlighted aspects to improve caregiver preparedness of stroke and its impact on care; health planning, recovery, and public health policies rarely consider these factors, reducing engagement and increasing uncertainty. Hence, there is a need to focus on improving strategies during recovery to promote caregiver engagement. In this study, we, therefore, try to understand the needs of the caregiver in stroke that limit engagement, and processes employed in countries such as Australia and Denmark to provide care for the person with stroke. Based on our understanding of these factors, we highlight the potential opportunities and challenges to promote caregiving engagement in these countries.

## Introduction

In the past few decades, there has been a shift in the overall global disease burden from infectious, nutritional, neonatal, and maternal causes to non-infectious diseases, with cardiovascular diseases and stroke being the predominant causes (1). Amongst cardiovascular disease and stroke, stroke remains a global health problem (2); as it is one of the leading causes of death and disability in the modern world (3). Recent data from the global burden of diseases study demonstrates that stroke accounts for 10% of deaths worldwide and 5% of disability-adjusted life-years (4). Moreover, the type of care provided to persons living with stroke over their lives depends on the type of stroke and its consequences (3). Over the years, ~3–4% of the total health expenditure in Western counties has been spent in stroke management and care. For example, the lifetime cost in the US for stroke inpatient care, rehabilitation and follow-up per person was estimated to be around US$ 140,048 (5). In Europe, the annual costs for stroke treatment and recovery were reported to be 27 billion euros (3). The increase in health care costs has resulted in the majority of care being conducted in the community rather than within health institutions (6) such as hospitals, rehabilitation centers etc. As a result, many family members, generally female spouses of the survivor with an average age of 58 years (7), take on the responsibility to become primary caregivers to people living with stroke (8) to ensure continuity of care.

The process of care in stroke is complex (9), and varies based on the needs, functional capabilities and support required by the person living with stroke (10). Most caregivers are often unprepared to assume their caregiving role immediately after stroke as it involves managing personal hygiene care, monitoring health and illness, administering medications, planning, and coordinating social activities and managing finances (11). Hence, several caregivers give up their dreams and aspirations to fulfill their new roles and responsibilities, which contributes to a significant burden (12). The burden of stroke caregiving is due to physical and financial strain, loneliness, confinement, and a myriad of mental and emotional strains that results in a negative health decline of the caregiver Camak (13). Furthermore, the disease is associated with long-term costs by the level of disability, with (14), estimating the cost per person in Spain to be around €17,618 per year, inclusive of informal care costs, medical costs and productivity-related costs. Another study by Taylor et al. (15) and Pucciarelli et al. (16) reported that individuals spent ~$3,700 on direct stroke-related (e.g., medical and non-medical) costs in Italy, with the highest cost incurred during the first 6 months of diagnosis. With most caregivers changing their work situations post-stroke, i.e., from full-time job to either a part-time job or leaving their job completely (17), there is a sudden loss of income due to the lack of work productivity. A previous study described the reduction in work productivity (18) amongst caregivers especially with older caregivers having children <18 years had resulted in a significant impact on the income levels (income < $25,000, *P* = 0.02; income between $25,000 and $49,999, *P* = 0.041 vs. those individuals with an income ≥75,000) (19). The loss of income and decreased work productivity makes it difficult to manage the financial aspects of stroke caregiving leading to additional stressors or burden (12).

Despite the burden involved in care, caregivers often want to be involved in recovery and provide tangible assistance and support for the person living with stroke (20). Engaging caregivers in the healthcare process is considered to be a key pillar in improving the effectiveness and sustainability of services (21). However, previous studies highlighted the lack of inclusion of caregivers in the recovery process; leading to the caregiver feeling neglected or abandoned by the healthcare team (22). Hence, there is a need to identify effective ways to engage the caregiver in the stroke recovery process to improve safety, quality and delivery of stroke care.

In an attempt to understand the process of engaging the caregiver in stroke within the community and recommend possible mechanisms to support the caregiver, we consider a multi-country perspective; including two developed countries (i.e., Denmark and Australia) with a publicly funded healthcare system (23). The process of stroke care in Denmark and Australia were reviewed as they have recently gained widespread public, political and academic interest for providing volunteer-based care in the community (24); with considerable differences. For example, in Denmark, people with stroke are provided professional and financial support for volunteer-based activities (25), which is not the same in Australia where people with stroke are often supported by the family members in coordination with the healthcare professional (26). Despite this difference, these volunteers may require support and training to manage the person living with stroke and perform self-support (27). Moreover, these countries allow for the individuals to access their health data; to promote self-management and care (28) that could be beneficial for long-term recovery of the patient and engagement for the caregiver (29).

## Caregiver Engagement in Stroke

Caregiver engagement refers to an active partnership between the patients, families, and health care providers at various levels to improve health outcomes (30), which is central for person-centered care (31). The caregiver engagement at direct level focuses on information seeking, consultation and involvement in decision making (21), while at an organization and societal level it focuses on shared leadership required to develop better health policies (32). Active engagement by the caregiver has the potential to reduce healthcare costs, reduce burnout, improve care processes and improve patient outcomes (30). Despite these advantages in transforming healthcare delivery and policy, very little is known about strategies to engage caregivers during the stroke care trajectory effectively. Hence, in this section, we identify means to improve caregiver engagement at different levels, i.e., planning, recovery and policymaking.

### Healthcare System Planning and Policy Making

Caregivers who support people affected by stroke often report proper support during in-patient care, but poor support post-discharge (33). Post-discharge, the caregivers, often feel unprepared and uncertain about the future; leading to poor health outcomes and reduced quality of care (22). Hartford et al. (34) have suggested that community services are not often coordinated efficiently. For example, in Denmark, caregivers reported a delay of up to 4 weeks in community services after discharge (35), while in Australia caregivers reported been uninformed about the care process during the transition from hospital to the community, and are unaware of the services available to them post-discharge (36); thereby impacting the continuity of care.

The healthcare system in Denmark and Australia is focused on detecting, monitoring, diagnosing, treating and providing care to individuals based on the public health policy (37, 38). These policies have been developed through collaboration with different stakeholders (i.e., patient and caregivers), community leaders and representatives from governments to solve social and community problems (31). At this level, it is necessary to define the concept of engagement to ensure priorities are defined, and the program makes informed decisions. According to Hill et al. (32), the concept of caregiver engagement should focus on understanding the role of the caregiver during recovery and individual factors that may contribute to the initiation of care and maintaining partnerships. By understanding these factors, it would be possible to create a plan that (i) supports individual characteristics of the patient, (ii) ensure the preparedness of the caregiver through the generation of knowledge and skills, and (iii) determining the capacity and preparedness of the recovery team to maintain care relationships. Furthermore, when defining the health policy, one would need to include the caregiver's desire to participate in community services and decision-making practices, which would inform the healthcare professionals to reach desired health outcomes.

Both Denmark and Australia have well-defined health policy guidelines for stroke recovery. In Denmark, the health policies were defined during a stroke care reform to centralize acute stroke care (39). Based on this reform, the long-term care was provided through a collaboration between the municipality rehabilitation centers and the hospital, with an intention to move rehabilitation care to the community and reduce healthcare costs during in-patient care (39). In 2012, the reform was modified to include policies related to administrative, management and coordinating factors (39). Furthermore, this model allowed for caregivers and patients to receive physical, emotional and social health support from the municipalities to reduce burden (40). In Australia, however, stroke care policies have undergone numerous different modifications over the past decade to support and integrate the different stakeholders in stroke recovery. Some of these reforms include the inclusion of appropriate education for caregivers, strategies to support impairments of the people living with stroke and inclusion of caregivers in stroke recovery (41). Despite the policies being implemented to support caregivers in stroke; the caregivers have reported burden due to the lack of unmet needs during recovery. These unmet needs include physical and emotional strain, isolation, emotional involvement and time spent on caregiving in Denmark (42), and social isolation, change in roles and relationships, and lack of services and support in Australia (43). These unmet needs can influence on the caregiver function resulting in reduced engagement in care (21). Hence, requiring for a clear understanding of the requirements of caregivers during the design of community services and policies in stroke recovery to ensure proper support, communication and engagement practices are employed.

**Figure 1:**
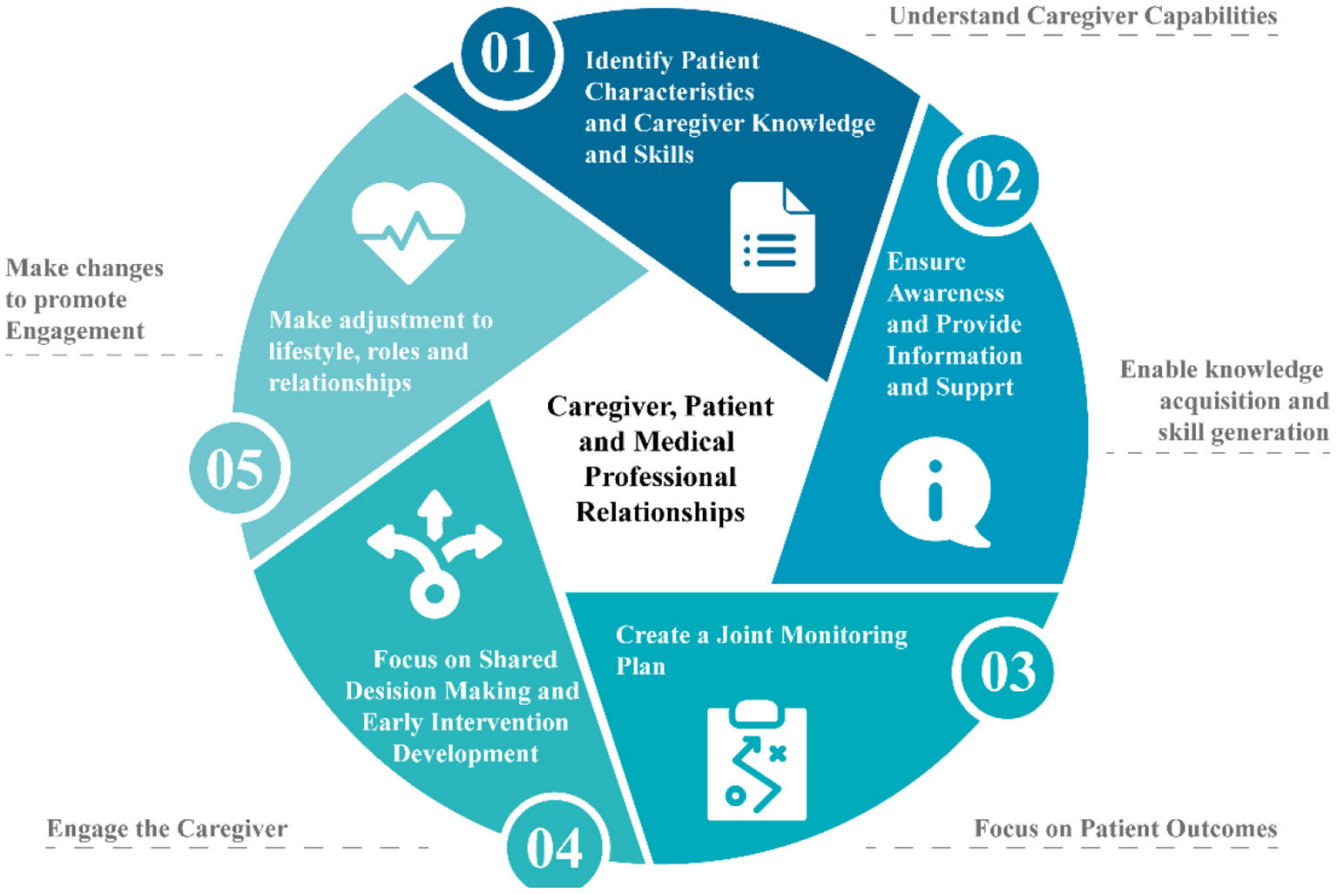
Model for defining caregiver engagement process in health planning and policy making.

Hill et al. (32) defined a model to identify caregiver engagement, as shown in Figure 1. This process includes negotiation and risk assessment, awareness and information support, joint monitoring plan, shared decision making and early intervention, and making adjustments. Through the inclusion of such a process, it is possible to engage the caregiver required for healthcare planning and policy making; allowing decision-makers to understand the needs and requirements of the caregivers in stroke recovery.

### Recovery

In countries such as Denmark and Australia, the median length of stay after acute stroke is between 3 and 7 days (26, 44). Rehabilitation occurs within the community in the form of rehabilitation services, outpatient facility or patent's home (39, 45). Community rehabilitation of stroke focuses on improving both immediate and long-term function of the person living with stroke to increase independence; irrespective of their age, stroke type, severity, and reoccurrence (26). In this process, clinicians are often required to ensure the recovery process meets the individual needs of the person living with stroke (46). However, the quality of care post-discharge is challenging to monitor for the clinicians (39). Hence, caregivers often assume the role of supporting the patient during the disease trajectory and communicating with the healthcare professional to ensure the quality of care is maintained throughout the process of recovery (46, 47).

Engaging caregivers in stroke throughout the disease trajectory can be challenging (48); as the rehabilitation and decision-making process requires the caregiver to have a certain degree of knowledge and understanding of the disease (49). But due to the abrupt nature of the disease; the caregiver is often left unprepared to manage the person living with stroke in the community (43). National organizations like the Department of Social Services, Department of Health and Aging, Department of Families, Community Services and Indigenous Affairs, Department of Human Services and the Department of Veterans Affairs in Australia, and the Municipality Care Services in Denmark have a longstanding commitment toward ensuring support for the caregiver during the continuity of care through the provision of different programmes and services (40, 50). In addition to the National organization; several non-governmental organizations have been founded mostly in Australia to support individuals during stroke recoveries such as the Stroke Foundation, Carers, Care Search, Carer Gateway and My Time Peer Support Groups in Australia. Despite an abundance of services to help the caregiver in the acquisition of skills and knowledge, a majority of the caregivers are unaware of these services, and hence would need to be informed (51) to ensure better decision making and healthcare delivery.

Further, to facilitate engagement in stroke recovery; caregivers would not only need to be informed about the disease, rehabilitation, and decision-making process but would also be required to understand the model of care to ensure optimal recovery for the person living with stroke (34). The model of care is crucial to provide long-term support in the community and secondary prevention of the disease (52). In Australia, the model of care has been defined in the Acute Stroke Clinical Care Standard (53). Based on this standard, the caregiver and the person living with stroke are provided with an individualized care plan that describes the process of care including rehabilitation goals, medicines, and lifestyle modification required to manage risk factors. Moreover, the caregivers and person living with stroke are provided with follow-up appointments and contact details for ongoing care services (54). However, in Denmark, the process of care follows a top-to-down approach where the hospital is considered as the primary decision-makers, and the municipality follows the guidelines stated by the hospital (39). While people living with stroke and their caregiver have reported satisfaction due to the availability of professional support in the first-year post-stroke based on the model of care in Denmark. They often feel disconnected with healthcare services, as the perceived needs are not fulfilled (55). Therefore, requiring for a more integrated pathway that considers a multi-disciplinary team, including caregivers and people living with stroke to ensure share values in coordinating work and successful care (39).

## Discussion and Implications

Findings from this perspective demonstrate both theoretical and practical implications. First, the importance of engagement in stroke caregiving has been defined as a means to promote better quality care in the community, while ensuring improved decision-making and satisfaction in care. However, this process would involve proper education, skill generation and communication to contribute to the recovery process (21). Traditionally, healthcare organizations are expected to provide support to the caregiver to facilitate development in these aspects that are evident in the literature based on Danish and Australian contexts (40, 50, 56). However, caregivers report being unaware of these services leading to uncertainty and isolation in care (51). Moreover, very little research has been conducted in stroke caregiving engagement to understand the influence of such factors on the activities of the caregiver during the recovery trajectory. Additionally, healthcare policies in Denmark and Australia for stroke, do not account for engagement of the caregiver, and thus results in an uncertainty amongst the caregiver and the person living with stroke.

This study, highlights the need to develop the evidence to support stroke caregiving engagement by addressing the possible factors affecting the caregiver at different stages of health planning, recovery, and policymaking. Additionally, a detailed understanding of the processes involved in stroke care, available services and individual needs and experiences to create a more practical approach toward engagement with whom.

### Challenges in Implementing Caregiver Engagement

Implementing caregiver engagement in stroke is not without its challenges. Lack or perceived benefit, time constraints, increased workload and lack of awareness are some of the most common challenges. These barriers can be avoided through proper education and training, which is crucial not only to provide engagement but to improve the quality of care. The major challenge, however, in implementing caregiver engagement in stroke recovery is the power shift that may exist from shifting care from the medical professionals to the caregiver; arising from the decision-making authority and knowledge between the different stakeholders. Ultimately, it would be dependent on the caregiver, medical professional, and community support team to form effective partnerships; thereby promoting better engagement. In addition to the challenge of shifting the decision-making process, there is a significant gap in the literature regarding the opportunities and methodologies to promote active engagement in stroke recovery. The limited guidance leads to constraints regarding the effective means to encourage engagement and ensure the practices implemented are meaningful for the different stakeholders. One possible solution would be to consider the generic guidelines available in the literature and tailor it based on the requirements of stroke recovery. However, this would require active collaboration and participation between the various stakeholders involved in recovery.

## Conclusion

Caregiver engagement in stroke that is targeted to the different levels of care have the potential to reduce unmet needs and promote interaction with medical professional in an on-going basis. However, this would require the formation of relationships between the various stakeholders in recovery. Hence, there is a need to include public health policies that can promote caregiver engagement; especially in countries such as Denmark and Australia, where the primary focus of stroke care occurs within the community. Furthermore, there is a need for theoretical and practical evidence to highlight the potential of caregiving engagement in improving quality of care outcomes for the person living with stroke.

## Data Availability Statement

The original contributions presented in the study are included in the article/supplementary material, further inquiries can be directed to the corresponding author/s.

## Author Contributions

EL initiated this study to identify the models of care in stroke recovery, the contributions of the various stakeholders in ensuring efficient care to the person living with stroke using evidence-based perspectives, and performed this study under the supervision of AF and SI. Further, EL drafted the manuscript that was revised by AF, SI, LR, PL, FK, MA, and JG. All authors approved the final version of the manuscript.

## Funding

This study was supported through doctoral scholarships from the School of Information Technology, Deakin University, and the Department of Public Health, University of Copenhagen. JG was supported by an Australian Research Council Laureate Fellowship FL190100035.

## Conflict of Interest

The authors declare that the research was conducted in the absence of any commercial or financial relationships that could be construed as a potential conflict of interest.

## Publisher's Note

All claims expressed in this article are solely those of the authors and do not necessarily represent those of their affiliated organizations, or those of the publisher, the editors and the reviewers. Any product that may be evaluated in this article, or claim that may be made by its manufacturer, is not guaranteed or endorsed by the publisher.
